# Atlanta Medical College

**Published:** 1857-01

**Authors:** 


### EDITORIAL AND MISCELLANEOUS.

### ATLANTA MEDICAL COLLEGE.

For the benefit of those who are anxious to know something of the progress of affairs in reference to this Institution, and in response to inquiries upon the subject of the building, we have to say, that it is progressing with such rapidity, and is already so far completed, as to justify us in assuring our friends and those who are contemplating an attendance upon the next course of lectures, that there is *no doubt*, that it will be in readiness at the commencement of the regular term in May.

The edifice will be one of an imposing appearance, large, and well adapted to the purpose for which it is designed, meeting at once the demand for space, and the liberal arrangements, in every particular, required for the accommodation of the large classes, which are in a very few years to occupy it; so clearly indicated, by the almost unprecedented success, which has attended its progress thus far.

Indeed, in reference to the latter point, we may say, without qualification, that no institution, within our knowledge, medical or other, has attained the same success, in the same period, either as to numbers who have been in attendance, or with regard to the character and position which has been acquired, in view of the many obstacles which have had to be encountered, and the little pecuniary assistance derived from any and every quarter; indeed, it may be said to be almost exclusively the result of private enterprise, and private capital, combined with an energy which we have never seen equalled, much less excelled, and which has been determined to know no failure, or to be satisfied with anything less than the fullest success.

From the first, it has been the intention of the founders of this School, that it should be of the first order; and we hesitate not to say, that in five years, from the date of its Charter, it will have taken a position, not heretofore occupied by any similar institution, (with a single exception) in the United States, in double the length of time, and this we say, not from any assumption of extraordinary ability on the part of the Faculty, but from the peculiar advantages which the institution possesses, from location, season of lectures, cheapness of living, and the determined and united purpose of those who control it.

In disclaiming, however, what might be construed into a ridiculous presumption, as to the qualifications of the Faculty, we can say in reference to all, (ourself excepted,) that they have been fully endorsed, as entirely competent to the discharge of the duties which they are called on respectively to perform; several of them are acknowledged to be gentlemen of the highest order of talents and attainments, and, for the youngest member of the Faculty, now in Europe, a most brilliant career is allowed, upon all hands, to be clearly indicated; and though the writer of this article readily admits himself to be "least of all," he yet hopes, by diligence and perseverance, to be able to discharge his duties in a respectable manner.

Before concluding this hasty article, we would again acknowledge our indebtedness to the profession of the South, who have given so decided and efficient a response to the first effort which has been made in this section of the Union, to keep our students of medicine at home, during the summer, and we beg to assure them, that our continued and earnest efforts will be, to show that their confidence has not been misplaced.

